# Cardiac Amyloidosis-Challenging Diagnosis and Unclear Clinical Picture

**DOI:** 10.3390/medicina57050450

**Published:** 2021-05-06

**Authors:** Sylwia Kozak, Krzysztof Ulbrich, Maciej Migacz, Krzysztof Szydło, Katarzyna Mizia-Stec, Michał Holecki

**Affiliations:** 1 Student Scientific Society at the Department of Internal, Autoimmune and Metabolic Diseases, School of Medicine, Medical University of Silesia, 40-752 Katowice, Poland; sylwiakozak@icloud.com (S.K.); krzysztofulbrich8@gmail.com (K.U.); 2 Department of Internal, Autoimmune and Metabolic Diseases, School of Medicine, Medical University of Silesia, 40-752 Katowice, Poland; maciek.migacz@gmail.com; 3 1st Department of Cardiology, School of Medicine in Katowice, Medical University of Silesia, 40-752 Katowice, Poland; krzysztofszydlo@gmail.com (K.S.); kmiziastec@gmail.com (K.M.-S.)

**Keywords:** amyloidosis, heart failure, transthyretin

## Abstract

Cardiac amyloidosis (CA) is a rare systemic disease determined by the extracellular deposition of amyloid protein in the heart. The protein can accumulate in any part of the heart: myocardium, vessels, endocardium, valves, epicardium and parietal pericardium. The types of CA include the following types: light chain (AL), amyloidosis AA (Amyloid A) and transthyretin (ATTR). The detection of specific subtypes remains of great importance to implement the targeted treatment. We present the case of a 65-year-old woman, who was admitted with severe deterioration of exercise capacity, a bilateral reduction of physiological vesicular murmur, ascites and edema of lower extremities. CA was suspected due to echocardiographic examination results, which led to further examination and final diagnosis. The aim of this study is to improve the disease awareness among clinicians and shorten the delay between the first symptoms and the diagnosis establishment resulting in a better outcome.

## 1. Introduction

Amyloidosis is a disease entity in which proteins of abnormal structure gather in tissues [1]. The biochemical classification of amyloidosis includes the division into three, most common types: amyloidosis AL (Light chain), amyloidosis AA (Amyloid A) and amyloidosis ATTR (Transthyretin) [2]. Type AL is sometimes connected with immunocytic dyscrasias, however type AA often develops secondary to chronic inflammation. Amyloidosis can be generalized and affect multiple organs or localized with amyloid deposits in one organ. Organs that may be affected include heart, kidneys, liver or spleen. Heart is one of the most frequently involved organs as it concerns up to 70% patients with AL type of amyloidosis and about 10% of patients with amyloidosis ATTR. There are two distinct forms of ATTR: acquired wild-type ATTR (ATTRwt), also known as senile amyloidosis and hereditary ATTR (ATTRm) [3]. AA type of amyloidosis very rarely affects the heart. Unfortunately, there are no specific manifestations of cardiac amyloidosis. In most patients, there are heart failure symptoms, such as dyspnoea, edema of lower extremities, hepatosplenomegaly or ascites. We can also distinguish some red flags for suspicion of cardiac amyloidosis, including carpal tunnel syndrome, peripheral neuropathy symptoms or pseudo-infarct pattern in the electrocardiographic examination, [4]. There is also a propensity to orthostatic hypotension, which can lead to syncope or pre-syncope syndromes [5]. The overall symptoms are associated with poor quality of life [6]. The diagnosis is difficult and time-consuming, due to non-specific symptoms and the necessity to exclude other causes of heart failure, moreover, sometimes specialized procedures are required to confirm the diagnosis, which may not be available in some cases.

## 2. Case Presentation

In July 2019, a 65-year-old woman was admitted to the Department of Internal Medicine due to severe deterioration of exercise capacity which has been gradually increasing since autumn 2018. There were also two episodes of syncope with body strains. In February 2019, the patient was hospitalized in the cardiology department, where a cardiac cause of the symptoms was excluded despite the increased serum level of NT-proBNP. In March 2019, she was hospitalized at the pulmonology ward, however despite comprehensive diagnostics, no diagnosis was made. At present admission physical examination showed a bilateral reduction of physiological vesicular murmur, ascites and edema of lower extremities. Laboratory tests showed an elevated serum level of NT-pro-BNP (5602 pg/mL) high sensitivity troponin T (81.57 ng/L) and creatinine. Electrophoresis of serum and urine proteins was performed in order to exclude diseases associated with hypergammaglobulinemia among others type AL amyloidosis and multiple myeloma. There was an increase in serum alpha 1 and 2 globulin with a simultaneous decrease in the gamma globulin fractions. Moreover, the albumin to globulin ratio and total protein concentration was normal. Urine electrophoresis did not detect immunoglobulin light chain. According to the results of electrophoresis, bone marrow biopsy was performed, which excluded multiple myeloma and other hematologic malignancies. 

**Figure 1:**
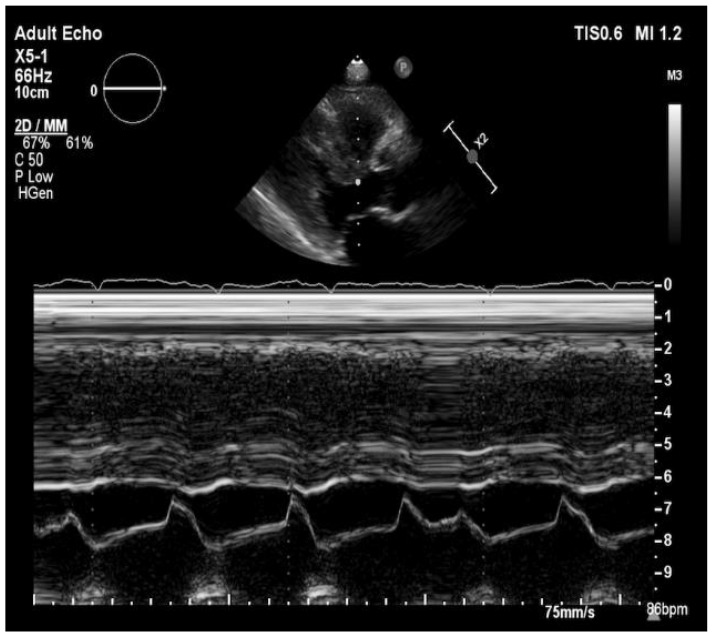
Transthoracic echocardiography in long parasternal view/M-Mode showing left ventricular hypertrophy.

The ultrasonography showed bilateral pleural effusion. MRI examination of pelvis confirmed massive edema of retroperitoneal and subcutaneous adipose tissue and inguinal lymphadenopathy. The echocardiographic examination (Figure 1) showed small dimensions of hearth cavities, the interventricular septum thickened to 22 mm, with the streaky brightening in the center, without significant valves disorders and right ventricle hypertrophy. The left ventricular end-systolic diameter was 12 mm and the left ventricular end-diastolic diameter was 24 mm, moreover, there was bilateral pleural effusion and 4 mm of fluid behind the left atrium. The left ventricle ejection fracture was preserved (55%). Electrocardiographic examination showed QS system in III, aVF, V1 and V2 and low QRS voltage in limb leads, Qtc was 503 ms.

The patient had two episodes of syncope, after which an electrocardiography examination revealed sinus bradycardia (HR 35/min) and prolongation of PQ interval. During hospitalization, the patients was provided with furosemide and eplerenone, and a low-dose of bisoprolol which resulted in improvement of patient’s condition, and a gradual regression of edema and the amount of fluid in pleural cavities. Due to an unclear clinical picture and the suspicion of amyloidosis the patient was referred to the cardiology department for further diagnosis.

**Figure 2:**
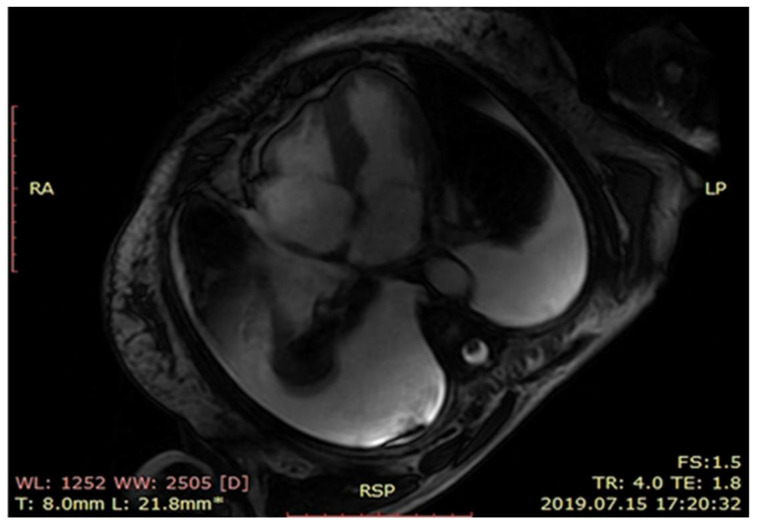
MRI scan showing left ventricle hypertrophy.

**Figure 3:**
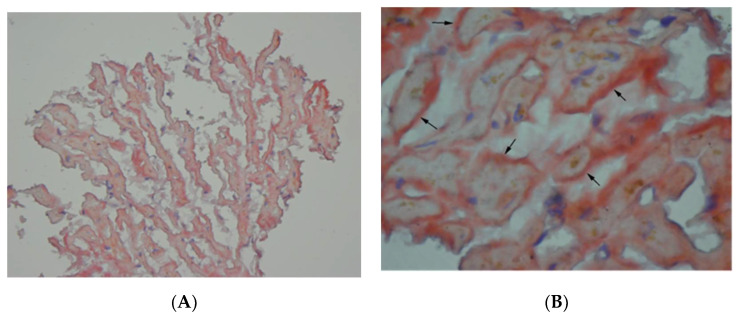
(**A**) Amyloid deposits and (**B**) amyloid deposits (arrows): red material in the section of myocardial biopsy stained with Congo red.

At cardiology department cardiac magnetic resonance (Figure 2) revealed left ventricle hypertrophy mainly concerning intraventricular septum, small ventricle dimensions and late gadolinium enhancement of the left ventricle muscle. Nuclear imaging with radiolabeled phosphonates (99mTc-DPD scintigraphy) was not available during the hospitalization thus to put the diagnosis a myocardial biopsy was done. The endomyocardial biopsy was complicated by a cardiac tamponade and the patient was immediately transferred to the cardio surgery operating theatre. Despite successful surgery, the intensive care unit treatment and an intravenous infusion of levosimendan, the patient died the next day after surgery. The histopathological examination including Congo red staining and evaluation of protein fibers in an electron microscope confirmed the diagnosis of a transthyretin type of amyloidosis (ATTR) (Figure 3A,B).

## 3. Discussion

In the presented case, despite the repeated hospitalizations, the diagnosis of ATTR was established with a long delay of 9 months. This might have been caused by various symptoms and the need to exclude other diseases. It confirms that amyloidosis is both rarely included in the differential diagnosis and the unclear clinical picture makes the diagnostic process time-consuming and complicated. Heart biopsy of an infiltrated myocardium is high risk procedure. Better availability of isotope scanning may limit severe complications of the invasive procedure [7,8]. However, in some cases the final diagnosis can only be made based on histopathological examination [9]. It can be of special importance for qualification for amyloidosis type-directed pharmacotherapy. 

The general CA treatment recommendations include the cardiac function support and the production as well as deposition of the precursor protein stopping [10] The loops diuretics and aldosterone antagonists are applied to manage heart failure and prevent excessive diuresis. As previously mentioned, the treatment targeting a specific subtype of amyloidosis results in a better disease prognosis and improves the patient’s quality of life. The ATTR drug-based treatment strategies are based on two processes: stabilization of the TTR tetramer or suppression of the TTR hepatic synthesis.

The TTR tetramer stabilizers include tafamidis and diflunisal. However, the first one is considered to be a better choice as its not associated with serious side effects [11], contrary to diflunisal causing severe kidney damage [12,13].

The TTR hepatic synthesis suppressors (the small interfering RNA patisiran and antisense oligonucleotide inotersen) both decrease the hepatic TTR synthesis and both are similar in effectiveness and tolerance [14].

## 4. Conclusions

Amyloidosis is a rare condition, although it should be considered in patients with unknown reason for heart failure, especially in cases with preserved ejection fraction [15], as its prevalence rate is still increasing. In our case report we aimed to describe a diagnostic work-up, clinical outcome of patient and a risk of endomyocardial biopsy. In our opinion, consideration of cardiac amyloidosis in the differentiation of causes of heart failure is very important, especially at the beginning of the onset of symptoms. The use of nuclear medicine techniques and cardiac magnetic resonance imaging can significantly contribute to early recognition of cardiac amyloidosis without exposing the patient to invasive diagnostic procedures [16].
